# Association between the body roundness index and osteoarthritis: evidence from NHANES

**DOI:** 10.3389/fmed.2024.1472196

**Published:** 2024-10-24

**Authors:** Tiancheng Ke, Jianqiang Lai, Xianmin Li, Fuqian Liu, Wei Liu, Chengfan Zhong

**Affiliations:** Department of Orthopedics, Gaozhou People’s Hospital, Maoming, China

**Keywords:** osteoarthritis, body roundness index, obesity, NHANES, cross-sectional study

## Abstract

**Background:**

The body roundness index (BRI) is a quantitative measure used to evaluate the presence of obesity and the distribution of body fat. However, the relationship between the BRI and osteoarthritis (OA) is still unclear. This study aimed to examine the relationship between the BRI and the occurrence of OA.

**Methods:**

This study was a cross-sectional analysis used to analyze data from the National Health and Nutrition Examination Survey (NHANES) from 2011 to 2018. A variety of variables were included in this investigation, which employed logistic regression analysis to assess the correlation between the BRI and OA. The robustness of the results and the impact of stratification variables were evaluated using subgroup and sensitivity analyses. To evaluate the ability of the BRI to predict OA, receiver operating characteristic (ROC) analysis was performed.

**Results:**

The analysis included 19,717 participants. Participants with OA had a significantly greater BRI than those without OA. Logistic regression analysis revealed a statistically significant positive correlation between the BRI and OA (OR = 1.18, 95% CI = 1.15–1.21, *p*-value <0.001). Despite the complete adjustment for covariates, this association remained stable (OR = 1.10, 95% CI = 1.04–1.17, *p*-value = 0.002). The results were corroborated by subgroup and sensitivity analyses, which demonstrated their robustness. Moreover, the BRI exhibited greater predictive accuracy for OA than did BMI.

**Conclusion:**

The BRI and OA are significantly associated in adults in the United States. The risk of developing OA may be increased by elevated levels of the BRI. Monitoring levels of the BRI is essential to prevent or reduce the prevalence and advancement of OA.

## 1 Background

Osteoarthritis (OA) is a complex degenerative disease that affects the entire joint and causes pathological changes in a number of joint structures, including cartilage, subchondral bone, synovium, ligaments, menisci, and surrounding muscles (1, 2). OA is a complex disease driven by multiple factors, of which aging and joint damage are recognized as major risk factors (3). At the same time, chronic diseases such as obesity, diabetes, and cardiovascular disease play an important role in the development and progression of OA by promoting systemic inflammation and metabolic disorders (1). OA causes chronic pain and limited mobility in patients, significantly impacting daily life and mental health (4, 5). From a societal perspective, the loss of labor and decreased productivity due to OA significantly impact the economy (6, 7). The prevalence of OA is anticipated to rise globally as a result of the growing number of elderly individuals, which will present a significant challenge to public health systems (8). In OA, the infrapatellar fat pad (IPFP), as an important adipose tissue in the joint, plays a dual role in the onset and development of OA with its inflammatory response and fibrosis, and its altered biomechanical properties are closely related to joint injury and cartilage degeneration (9, 10). Given the unique role of the infrapatellar fat pad in OA, evaluating the relationship between obesity and the prevalence of OA is critical for the prevention and effective management of OA.

Over the past few years, there has been an increasing interest in the relationship between obesity and OA (11–13). Obesity increases the mechanical load on the joints, especially the knee, leading to increased cartilage wear and tear (14). Furthermore, the systemic low-grade inflammation that accompanies obesity is a key factor in OA, and pro-inflammatory factors secreted by adipose tissue, such as IL-6 and TNF-*α*, exacerbate cartilage damage and joint inflammation (15). Obesity also leads to macrophage infiltration in adipose tissue, further exacerbating local inflammation and driving the pathological process of OA (16). Body mass index (BMI) is frequently used to screen for weight issues and to identify obese individuals (17). However, BMI has limitations because it ignores the heterogeneity of fat distribution and cannot differentiate between muscle and fat mass (18). The body roundness index (BRI) was first proposed by Thomas et al. in 2013, aiming to assess an individual’s body fat distribution, especially abdominal fat, through simple height and waist circumference measurements (19). Unlike the traditional BMI, the BRI not only takes into account body weight but also more accurately reflects the shape and fat distribution of the body and is particularly sensitive in assessing the risk associated with abdominal obesity (20). The BRI has been widely used in a variety of studies to predict the risk of a number of chronic diseases, including cardiovascular disease, metabolic syndrome, depression, and cancer (21–23). Specifically, a cross-sectional study found a significant positive correlation between the prevalence of depression and BRI levels (23). Another study further showed a significant positive correlation between BRI levels and the prevalence of colorectal cancer, emphasizing the importance of controlling BRI levels for the prevention of colorectal cancer (21). In addition, the BRI levels have been rising in U.S. adults over the past 20 years, and studies have shown a U-shaped relationship between the BRI and the risk of all-cause mortality, suggesting that either too high or too low BRI levels may increase the risk of death (20). However, the precise correlation between the BRI and OA remains unclear. Therefore, this study utilized NHANES survey data from 2011 to 2018 to explore the association between the BRI and OA. This study aimed to establish an empirical foundation for future public health interventions and to provide new evidence that substantiates the application of the BRI in OA.

## 2 Methods

### 2.1 Study design

The National Health and Nutrition Examination Survey (NHANES) is a research study that evaluates the health and nutritional status of the U.S. population. It does this by using a sampling method that is multistage, complicated, and stratified. To guarantee the accuracy and representativeness of the sample, the investigation implements a sophisticated multistage probability sampling methodology. The NHANES data are all publicly available.^1^ The study was approved by the National Center for Health Statistics (NCHS) Research Ethics Review Board (ERB) to ensure compliance with ethical guidelines for research involving human participants, and all participants provided informed consent forms.

### 2.2 Study population

This investigation employed data from the NHANES 2011–2018 cycles, which covered four cycles. The inclusion criteria were as follows: (1) individuals who were over 20 years of age, (2) individuals who had complete arthritis data, and (3) individuals who had complete BRI data.

### 2.3 Calculation of BRI

The BRI is calculated by combining two key body measurements: height (BH) and waist circumference (WC). Professionally educated medical technicians gathered the measurements at a mobile examination center (MEC). The formula to calculate the BRI is as follows:


$$BRI=364.2-365.5\times \sqrt{1-{\left(\frac{WC\left(m\right)/2\pi }{0.5\times BH\left(m\right)}\right)}^{2}}$$

### 2.4 Definition of OA

OA was assessed using a self-report form. Participants were initially queried, ‘Has a physician ever diagnosed you with arthritis?’ Respondents who said yes were then asked a follow-up question: ‘What type of arthritis?’ Individuals who specified OA or degenerative arthritis were classified as having OA, whereas all other responses were categorized as non-OA. Relevant investigations have established the validity of self-reported OA history (24, 25).

### 2.5 Covariates

This study included a range of covariates, such as demographic characteristics, lifestyle factors, health status, and laboratory test indicators. The demographic parameters included age, sex, ethnicity, BMI, and education level. Lifestyle variables included smoking, alcohol consumption, and physical activity levels. Smoking status was determined through a questionnaire, with individuals who had consumed more than 100 cigarettes in their lifetime classified as smokers. Physical activity levels were calculated based on metabolic equivalents (METs) according to guidelines, with MET values of less than 600 min per week defined as inactive (26). METs were calculated as follows: MET (min/week) = MET value × frequency (times/week) × duration (minutes/session). Health status was determined by doctor diagnosis or self-report and included conditions such as diabetes, coronary artery disease (CAD), and chronic kidney disease (CKD). Laboratory indicators included blood uric acid, blood urea nitrogen, alanine transaminase (ALT), aspartate transaminase (AST), high-density lipoprotein (HDL), and total cholesterol (TC).

### 2.6 Statistical analysis

This study included four survey cycles of the NHANES database from 2011 to 2018 for cross-sectional analyses. Participants displayed baseline characteristics according to OA status, and differences in baseline characteristics were assessed between the two groups. Stepwise model-adjusted logistic regression analyses were used to examine the association between the BRI and OA prevalence. Model I: There was no adjustment for covariates. Model II: adjusted for age, sex, and race. Model III: adjusted for age, sex, race, body mass index, smoking, alcohol consumption, physical activity, CAD, CKD, diabetes mellitus, blood calcium, blood phosphorus, SUA, BUN, ALT, AST, HDL, and TC. In addition, the BRI was categorized from a continuous variable into four categories (Q1: <3.92; Q2: 3.92–5.20; Q3: 5.20–6.82; Q4: >6.82) to explore the association trend between BRI levels and OA, thereby improving the robustness of the results. Restricted cubic spline curve (RCS) analyses revealed the association between the BRI and OA prevalence. Subgroup analyses investigated potential factors underlying the association between the BRI and OA prevalence, such as demographic characteristics and lifestyle habits. Sensitivity analyses were also performed to confirm the consistency and robustness of the findings. Finally, receiver operating characteristic (ROC) analyses were performed to assess the predictive power of the BRI for OA. Statistical significance was defined as *p* < 0.05, and all analyses were performed using R software (version 4.2.3).

## 3 Results

### 3.1 Baseline characteristics

**Figure 1:**
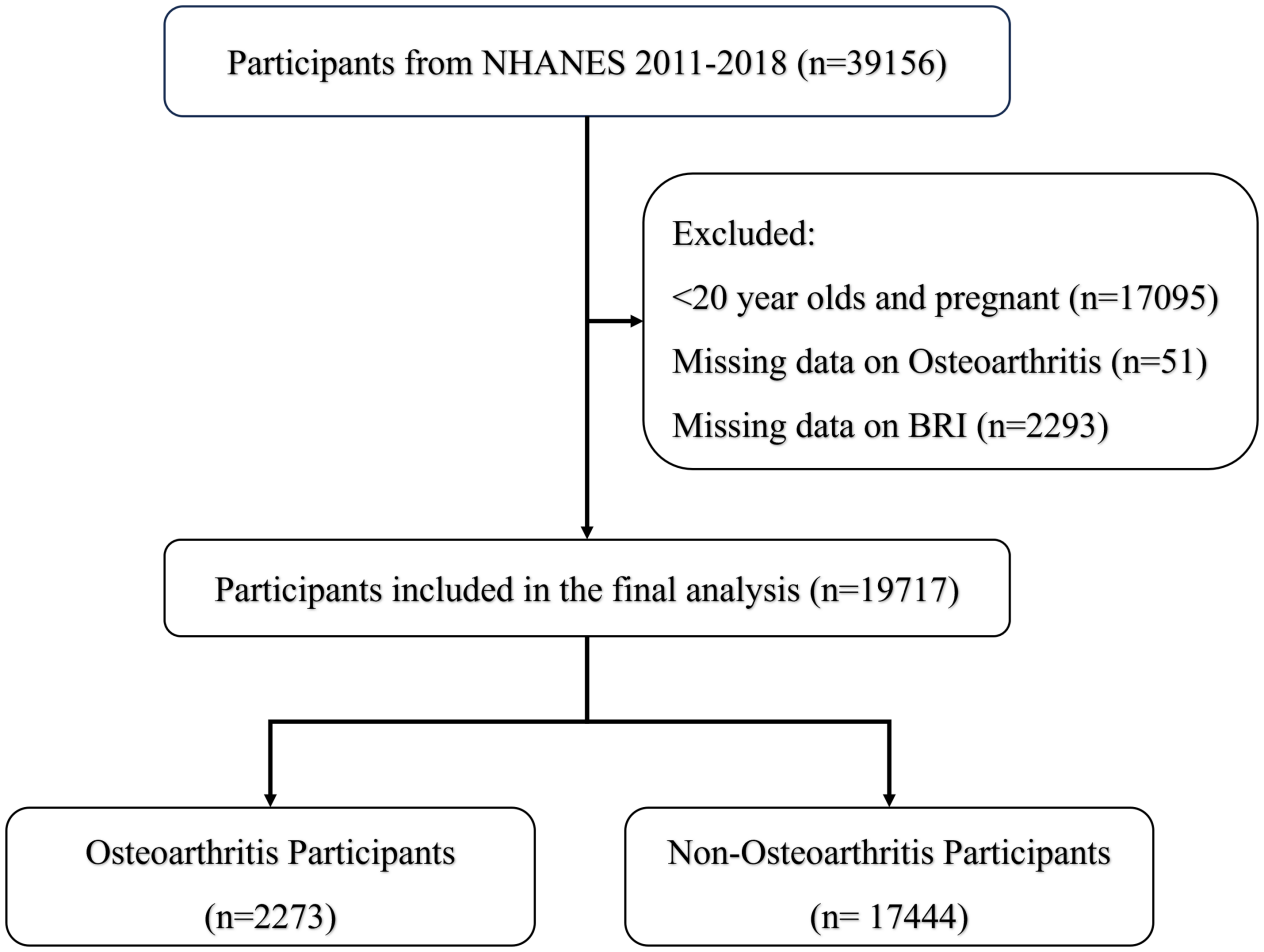
Participants involved in the process.

Figure 1 shows data from 19,717 participants extracted from the NHANES database, including 17,444 non-OA participants and 2,273 OA participants. The characteristics of the participants were categorized according to the presence or absence of OA, as detailed in Table 1, and weighted baseline characteristics are shown in Supplementary Table S1. Participants with OA were generally older, predominantly female, non-Hispanic white, and obese and exhibited lifestyle behaviors such as smoking and inactivity, compared to participants without OA. In addition, these patients had higher levels of HDL, TC, BUN, and WC. The prevalence of diabetes, CAD, and CKD was also higher in patients with OA. Notably, patients with OA had higher BRI levels, suggesting a possible correlation between high BRI levels and the prevalence of OA.

**Table 1:** Baseline characteristics of the study population.

Characteristic	Overall	Non-OA	OA	*p*-value
*n*	19,717	17,444	2,273	
Age (%)				<0.001
<50	10,082 (51.1)	9,694 (55.6)	388 (17.1)	
>50	9,635 (48.9)	7,750 (44.4)	1885 (82.9)	
Sex (%)				<0.001
Female participants	10,038 (50.9)	8,587 (49.2)	1,451 (63.8)	
Male participants	9,679 (49.1)	8,857 (50.8)	822 (36.2)	
Ethnicity (%)				<0.001
Mexican-American	2,665 (13.5)	2,480 (14.2)	185 (8.1)	
Non-Hispanic Black	4,457 (22.6)	4,069 (23.3)	388 (17.1)	
Non-Hispanic White	7,281 (36.9)	5,978 (34.3)	1,303 (57.3)	
Other	5,314 (27.0)	4,917 (28.2)	397 (17.5)	
BMI (%)				<0.001
Normal	5,745 (29.2)	5,276 (30.3)	469 (20.7)	
Overweight	6,368 (32.3)	5,700 (32.7)	668 (29.4)	
Obese	7,582 (38.5)	6,448 (37.0)	1,134 (49.9)	
Education level (%)				0.001
Less than high school	4,296 (21.8)	3,873 (22.2)	423 (18.6)	
High school	4,357 (22.1)	3,854 (22.1)	503 (22.1)	
Above high school	11,051 (56.0)	9,706 (55.6)	1,345 (59.2)	
No record	13 (0.1)	11 (0.1)	2 (0.1)	
Smoke (%)				<0.001
No	11,215 (56.9)	10,126 (58.0)	1,089 (47.9)	
Yes	8,489 (43.1)	7,306 (41.9)	1,183 (52.0)	
No record	13 (0.1)	12 (0.1)	1 (0.0)	
Alcohol consumption (%)				0.411
No	4,320 (23.6)	3,829 (23.8)	491 (22.5)	
Yes	13,958 (76.3)	12,271 (76.2)	1,687 (77.4)	
No record	12 (0.1)	11 (0.1)	1 (0.0)	
Activity status (%)				<0.001
Active	10,411 (52.8)	9,459 (54.2)	952 (41.9)	
Inactive	9,306 (47.2)	7,985 (45.8)	1,321 (58.1)	
Diabetes (%)				<0.001
No	16,453 (83.4)	14,806 (84.9)	1,647 (72.5)	
Yes	2,725 (13.8)	2,206 (12.6)	519 (22.8)	
No record	539 (2.7)	432 (2.5)	107 (4.7)	
CKD (%)				<0.001
No	19,013 (96.4)	16,903 (96.9)	2,110 (92.8)	
Yes	682 (3.5)	523 (3.0)	159 (7.0)	
No record	22 (0.1)	18 (0.1)	4 (0.2)	
CAD (%)				<0.001
No	18,369 (93.2)	16,448 (94.3)	1921 (84.5)	
Yes	1,348 (6.8)	996 (5.7)	352 (15.5)	
SUA (mg/dL)	5.44 (1.44)	5.44 (1.43)	5.49 (1.45)	0.135
HDL (mmol/L)	1.37 (0.42)	1.37 (0.41)	1.43 (0.45)	<0.001
TC (mmol/L)	4.93 (1.07)	4.92 (1.06)	5.00 (1.15)	0.001
BUN (mmol/L)	5.02 (2.12)	4.93 (2.05)	5.73 (2.50)	<0.001
ALT (U/L)	24.64 (20.59)	24.85 (21.25)	23.06 (14.51)	<0.001
AST (U/L)	24.89 (16.92)	24.92 (17.32)	24.72 (13.45)	0.606
Calcium (mmol/L)	2.34 (0.09)	2.34 (0.09)	2.35 (0.10)	0.006
Phosphorus (mmol/L)	1.19 (0.18)	1.19 (0.18)	1.21 (0.18)	<0.001
WC (cm)	99.80 (16.58)	99.04 (16.36)	105.61 (17.07)	<0.001
BH (cm)	166.80 (10.13)	167.05 (10.11)	164.93 (10.09)	<0.001
BMI (kg/m^2^)	29.28 (6.95)	29.02 (6.79)	31.25 (7.80)	<0.001
BRI	5.61 (2.40)	5.48 (2.34)	6.60 (2.62)	<0.001
BRI (%)				<0.001
Q1	4,930 (25.0)	4,654 (26.7)	276 (12.1)	
Q2	4,930 (25.0)	4,431 (25.4)	499 (22.0)	
Q3	4,927 (25.0)	4,327 (24.8)	600 (26.4)	
Q4	4,930 (25.0)	4,032 (23.1)	898 (39.5)	

Mean (SD) for continuous variables and % for categorical variables. ALT, alanine aminotransferase; AST, aspartate aminotransferase; BH, body height; BMI, body mass index; BRI, body roundness index; BUN, blood urea nitrogen; CAD, coronary artery disease; CKD, chronic kidney disease; SUA, serum uric acid; TC, total cholesterol; TG, triglyceride; WC, waist circumference.

### 3.2 Association between the BRI and OA prevalence

**Figure 2:**
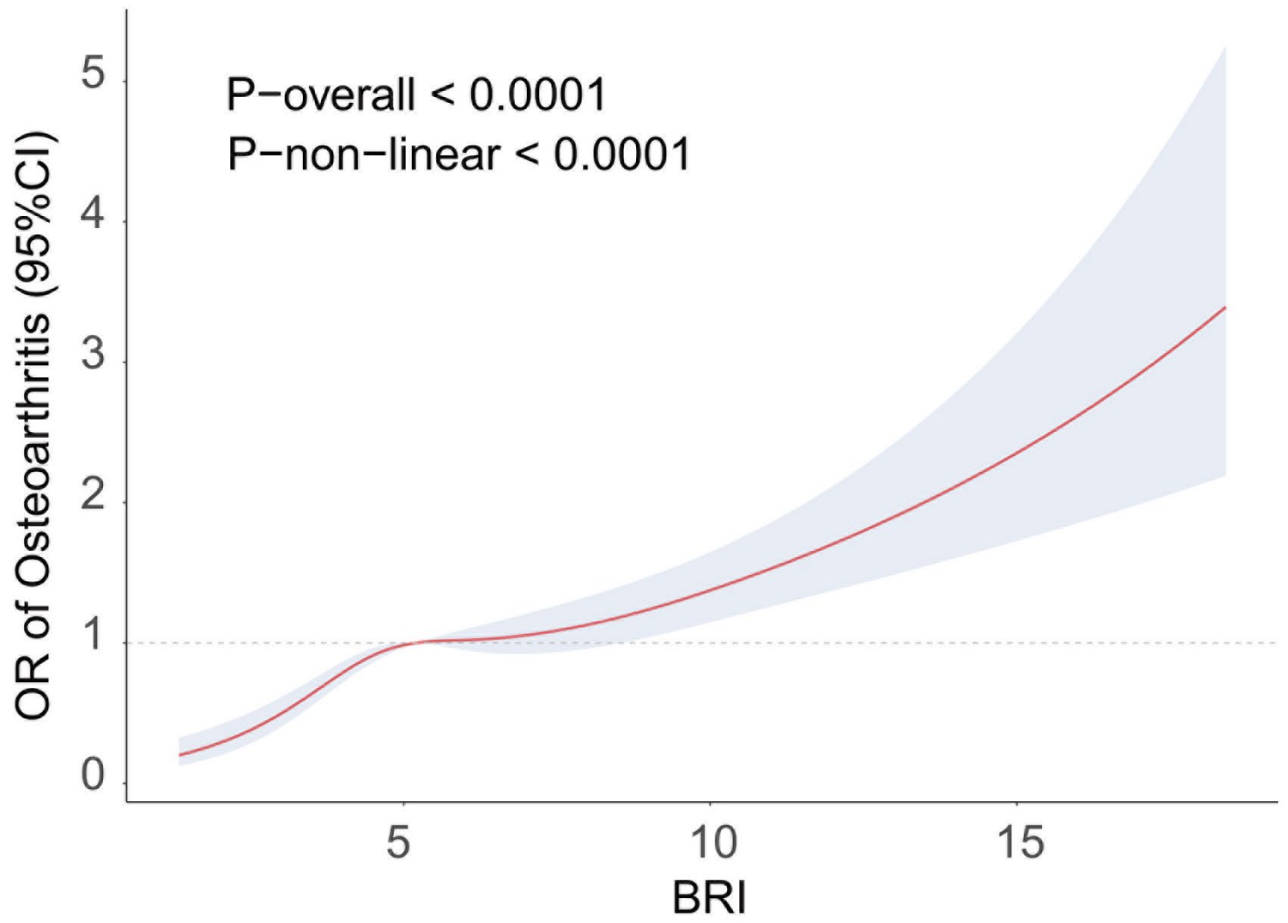
RCS curve fitting the association of the BRI with osteoarthritis. Adjusted for age, sex, ethnicity, BMI, education level, smoking, alcohol consumption, activity status, CAD, CKD, diabetes, calcium, phosphorus, SUA, BUN, ALT, AST, HDL, and TC.

Table 2 presents the relationship between the BRI and OA prevalence as analyzed using logistic regression. In Model 1, a significant relationship between the BRI and OA prevalence was found (OR = 1.18, 95% CI = 1.15–1.21, *p*-value <0.001). After progressively adjusting for various covariates, Model 3 still indicated a significant positive correlation between the BRI and OA prevalence (OR = 1.10, 95% CI = 1.04–1.17, *p*-value = 0.002). Further analysis of BRIs categorized into quartiles revealed that increasing BRIs were significantly associated with increased OA prevalence (P trend<0.001). The highest quartile of the BRI remained significantly associated with a greater prevalence of OA, even after adjusting for all covariates (OR = 1.78, 95% CI = 1.24–2.54, *p*-value = 0.003). Figure 2 illustrates a non-linear relationship between the prevalence of OA and the BRI, as demonstrated by the RCS analysis. These results suggest a strong positive correlation between the BRI and OA.

**Table 2:** Relationship between the BRI and OA.

		Model 1 OR (95%CI) *p*-value	Model 2 OR (95%CI) *p*-value	Model 3 OR (95%CI) *p*-value
OA	BRI	1.18 (1.15, 1.21) <0.001	1.11 (1.06, 1.16) <0.001	1.10 (1.04, 1.17) 0.002
	Q1	[Reference]	[Reference]	[Reference]
	Q2	2.06 (1.67, 2.54) <0.001	1.60 (1.27, 2.01) <0.001	1.59 (1.20, 2.10) 0.002
	Q3	2.51 (2.09, 3.02) <0.001	1.75 (1.45, 2.13) <0.001	1.51 (1.15, 1.99) 0.004
	Q4	3.67 (3.12, 4.31) <0.001	2.42 (2.02, 2.89) <0.001	1.78 (1.24, 2.54) 0.003
	P for trend	<0.001	<0.001	0.024

CI, confidence interval; OR, odds ratio; Q, quartiles; BRI, body roundness index.

Model 1, no covariates adjusted; Model 2, adjusted for age, sex, and ethnicity; Model 3, adjusted for age, sex, ethnicity, BMI, education level, smoking, alcohol consumption, activity status, CAD, CKD, diabetes, calcium, phosphorus, SUA, BUN, ALT, AST, HDL, and TC.

### 3.3 Subgroup and sensitivity analyses

**Figure 3:**
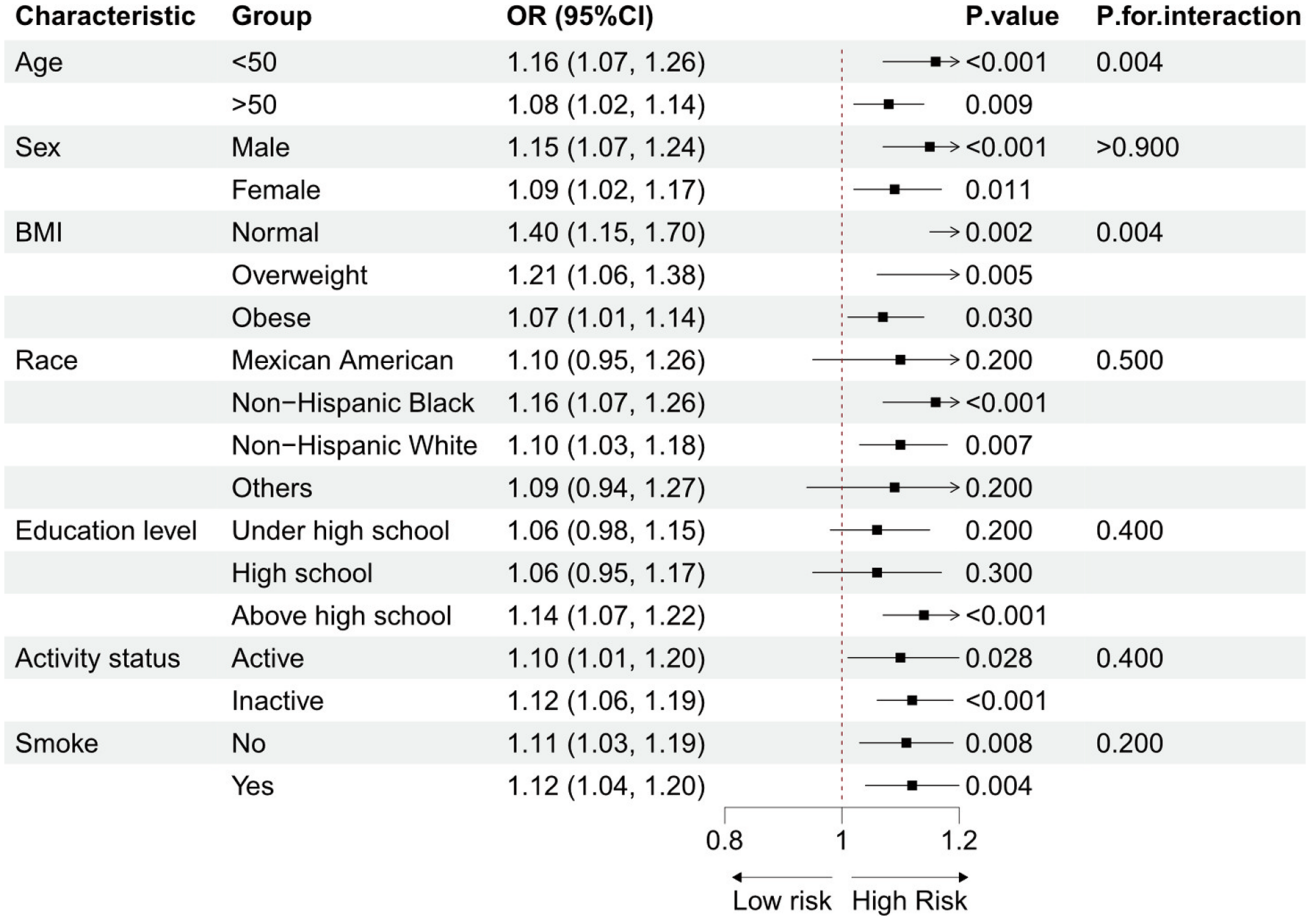
Subgroup analysis of the association between the BRI and osteoarthritis.

Subgroup analyses were conducted to explore the potential link between the BRI and OA, considering demographic characteristics and lifestyle factors (Figure 3). The results consistently showed a positive correlation between the BRI and OA prevalence across all groups, with no statistically significant interaction effects. This finding supports the notion that the BRI is an independent risk factor for OA. Supplementary Table S1 reveals the results of sensitivity analyses after excluding 7,582 obese subjects. The remaining 12,135 participants included 10996 non-OA participants and 1139 OA participants. The results of the sensitivity analyses were consistent with the primary results, further confirming a positive association between the BRI and OA. The findings of the sensitivity analyses aligned with the primary results, reinforcing the conclusion of a positive association between the BRI and OA.

### 3.4 Comparison of the BRI and the BMI in predicting OA

**Figure 4:**
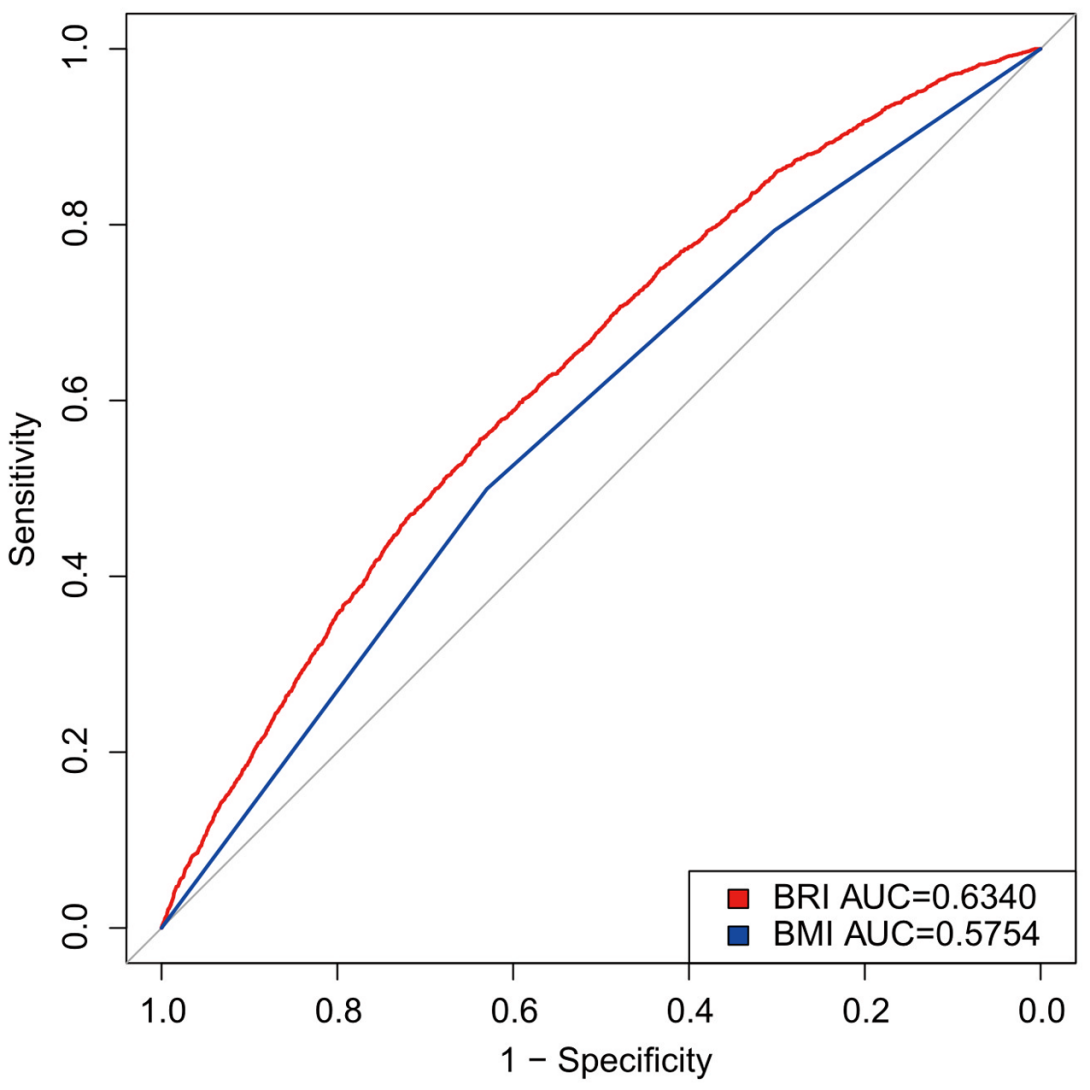
Diagnostic performance of obesity BRI and BMI index on OA prevalence.

The predictive efficacy of the BRI and the BMI for OA was evaluated by calculating the area under the ROC curve (Figure 4). The results showed that the AUC for the BRI (0.6340) was higher than that for the BMI (0.5754). Supplementary Table S2 shows that the BRI outperformed the BMI in both sensitivity and specificity, with a lower optimal threshold. These findings indicate that the BRI outperforms the BMI in its ability to predict OA.

## 4 Discussion

The present study was a cross-sectional analysis using the NHANES database designed to assess the association between the BRI and OA prevalence among U.S. adults. The results showed a significant positive association between high BRI levels and OA prevalence, which was consistently confirmed across subgroups. In addition, the efficacy of the BRI in predicting OA was found to be superior to that of traditional BMI in this study, which suggests that the BRI can be used as an effective tool for assessing the prevalence of OA, which is an important reference value for early clinical prevention and intervention.

Recently, the association between obesity and OA has garnered widespread attention, as the prevalence of both conditions has significantly increased globally (8, 27). In addition to increasing the likelihood of cardiovascular disease, diabetes, and specific malignancies, obesity also has a detrimental impact on bone and joint health (28–30). The progression of OA is significantly influenced by adiposity, as demonstrated by a multitude of studies (12, 31, 32). A Mendelian randomized study showed a positive genetic correlation between BMI and OA (33). Another study showed that increased BMI is an important risk factor for OA and increases the prevalence of OA (34). These findings are consistent with the results of the present study and further emphasize the key role of obesity in OA. The results of the present study showed that the BRI has higher sensitivity and specificity than the BMI in predicting OA, and in particular, the BRI is more sensitive to the assessment of abdominal adiposity, which is an important contributor to inflammation and metabolic abnormalities that may accelerate the onset and progression of OA (35, 36). This study also verified that the BRI has greater sensitivity and specificity than the BMI in predicting OA. Subgroup analyses indicated that the association between the BRI and OA remained significant across various age and sex groups, suggesting that the BRI may serve as a universal indicator for assessing OA. These results offer new insights into the early prevention and intervention of OA and present a more comprehensive approach to managing obesity-related diseases.

The pathophysiological mechanisms linking the BRI and OA involve multiple complex and interrelated factors. First, increased mechanical load is a significant factor through which obesity leads to OA. Increased body weight in obese individuals results in greater mechanical pressure on weight-bearing joints, especially the knees and hips, accelerating joint cartilage wear and degeneration (37, 38). In addition, mechanical load not only directly damages the cartilage matrix but also alters the inflammatory state of chondrocytes (39). Obesity-induced systemic inflammation is a critical component in the pathogenesis of OA (11). In obese individuals, adipocytes release significant quantities of pro-inflammatory cytokines such as interleukin-6 (IL-6) and tumor necrosis factor-alpha (TNF-*α*), which intensify joint inflammation and cartilage damage (40). Obesity is often accompanied by metabolic syndrome, including hyperglycemia, hypertension, and hyperlipidemia, which also adversely affect joint health (13, 41, 42). For instance, hyperglycemia promotes the formation of advanced glycation end products, which harm chondrocytes and the extracellular matrix (43). In summary, the relationship between the BRI and OA results from multifactorial and multi-pathway interactions, and further research into these mechanisms may aid in developing more effective prevention and treatment strategies.

This study has several significant strengths. First, the study used nationally representative NHANES data, which ensured the broad applicability and reliability of the results through weighted analyses. The study also incorporated multiple covariates, effectively controlling for potential confounders. However, there are some limitations to this study. Due to the cross-sectional design, it was not possible to determine the causal relationship between the BRI and OA. In addition, the diagnosis of OA relies on patient self-report, which may introduce information bias. The lack of specific imaging or clinical diagnostic data may not accurately reflect the true prevalence of OA. The present study also lacked data on the site of OA prevalence (e.g., knee, hip, and hand joints) and its severity, limiting in-depth exploration of the association between the BRI and different types and severities of OA. In addition, questionnaires that assess the level of pain and its impact on daily life in patients with OA were not included in this study, making it difficult to fully assess the impact of OA on patients’ quality of life. Future studies should consider incorporating assessment tools for pain and activities of daily living to explore the relationship more fully between the BRI and OA. To further confirm the results of this study, larger longitudinal studies should be conducted in the future, and the accuracy of the data should be improved by introducing imaging or clinical examinations.

## 5 Conclusion

The BRI and OA are significantly associated in adults in the United States. The risk of developing OA may be increased by elevated BRI levels. Monitoring BRI levels is essential to prevent or reduce the prevalence and advancement of OA.

## Data Availability

Publicly available datasets were analyzed in this study. This data can be found here: https://www.cdc.gov/nchs/nhanes/nhanes_products.htm.

## Footnotes

^1^ https://www.cdc.gov/nchs/nhanes/nhanes_products.htm
