# Supplementary material for: Association between the body roundness index and osteoarthritis: evidence from NHANES
Source: Front Med (Lausanne). 2024 Oct 24;11:1472196. doi: 10.3389/fmed.2024.1472196 (PMC11540616; doi:10.3389/fmed.2024.1472196)
Supplement: Supplementary file 1 [file Table_1.DOCX]

**S1 Table: Sensitivity analysis between BRI and OA.**

|  |  | Model 1  OR (95%CI) P-value | Model 2  OR (95%CI) P-value | Model 3  OR (95%CI) P-value |
| --- | --- | --- | --- | --- |
| OA | BRI | 1.53 (1.45, 1.62) <0.001 | 1.21 (1.13, 1.30) <0.001 | 1.28 (1.17, 1.41) <0.001 |
|  | Q1 | [Reference] | [Reference] | [Reference] |
|  | Q2 | 2.15 (1.74, 2.65) <0.001 | 1.56 (1.23, 1.96) <0.001 | 1.65 (1.24, 2.20) 0.001 |
|  | Q3 | 2.93 (2.41, 3.55) <0.001 | 1.53 (1.22, 1.91) <0.001 | 1.58 (1.18, 2.13) 0.004 |
|  | Q4 | 6.84 (4.61, 10.1) <0.001 | 2.52 (1.62, 3.90) <0.001 | 2.62 (1.52, 4.50) 0.001 |
|  | P for trend | <0.001 | <0.001 | 0.002 |

CI: Confidence Interval; OR: Odds Ratio; Q: Quartiles; BRI: Body Roundness Index

Model 1: No covariates adjusted; Model 2: Adjusted for Age, Sex, and Race; Model 3: Adjusted for age, Sex, Race, BMI, Educational level, Smoke, Drinking, Activity status, CAD, CKD, Diabetes, Calcium, Phosphorus, SUA, BUN, ALT, AST, HDL, TC.

**S2 Table: Comparison of ROC curves for BRI and BMI in predicting Osteoarthritis**

|  | AUC (95% CI) | Threshold | Sensitivity | Specificity |
| --- | --- | --- | --- | --- |
| BRI | 0.6340 (0.6222-0.6457) | 0.1333548 | 0.5561427 | 0.6374541 |
| BMI | 0.5754 (0.5639-0.5869) | 0.1476494 | 0.4993395 | 0.6299357 |
